# Successful treatment of lupus anticoagulant hypoprothrombinemia syndrome with rituximab

**DOI:** 10.1186/s12959-023-00517-z

**Published:** 2023-07-17

**Authors:** Sanober Nusrat, Sayani Tewari, Osman Khan

**Affiliations:** 1 Hematology-Oncology Section, Department of Medicine, University of Oklahoma Health Sciences Center, 800 NE 10th Street, Oklahoma City, OK 73104 USA; 2 Nemours Children’s Health, Seaford, Delaware, USA; 3 Pediatric Hematology-Oncology Section, Department of Pediatrics, University of Oklahoma Health Sciences Center, Oklahoma City, OK USA

**Keywords:** Lupus anticoagulant hypoprothrombinemia syndrome, Rituximab, Anti-CD20 monoclonal antibody, Thrombosis, Hemostasis

## Abstract

Lupus anticoagulant-hypoprothrombinemia syndrome (LAHPS) is a rare acquired bleeding disorder secondary to development of antibodies against prothrombin protein, in the presence of antiphospholipid antibodies. We describe the case of a 13-year-old girl who presented with severe menorrhagia and symptomatic anemia. Labs indicated anemia, thrombocytopenia, elevated PT and aPTT, high-titer inhibitor on mixing studies, positive ANA and anti-dsDNA antibodies, along with a triple-positive antiphospholipid antibody panel. Given additional systemic manifestations, systemic lupus erythematosus was diagnosed. High dose steroids and hydroxychloroquine subsequently started. Her clinical course was complicated by femoral deep venous thrombosis and post renal biopsy retroperitoneal hematoma. Further workup revealed low prothrombin level and the diagnosis of lupus anticoagulant hypoprothrombinemia syndrome. In view of suboptimal response to initial immunosuppressive therapy, rituximab was added to her regimen, leading to an improvement in clinical symptoms and resolution of hypoprothrombinemia. She remains recurrence free 5 years from the event.

**Supplementary Information:**

The online version contains supplementary material available at 10.1186/s12959-023-00517-z.

## Highlights

Lupus anticoagulant hypoprothrombinemia syndrome (LAHPS) is a rare acquired disorder caused by the formation of prothrombin antibodies in patients with antiphospholipid antibodies.

No standard of care exists due to paucity of data; treatment largely relies on supportive transfusions and immunosuppressive therapy.

We discuss the case of a 13-year-old girl with LAHPS who was successfully treated with rituximab after proven to be steroid-refractory.

LAHPS patients who demonstrate poor response to high-dose corticosteroids can potentially be treated with rituximab with good outcomes.

**Supplementary Information:**

The online version contains supplementary material available at 10.1186/s12959-023-00517-z.

## Background

Lupus anticoagulant hypoprothrombinemia syndrome (LAHPS) is a rare acquired disorder caused by prothrombin antibodies in the presence of lupus anticoagulant. Severe life-threatening bleeding is the hallmark of LAHPS, which in the pediatric population, most commonly occurs in association with underlying systemic lupus erythematosus (SLE) or a viral infection [1].

## Case presentation

We present the case of a 13-year-old girl who presented to the emergency department with severe fatigue and pallor. She reported excessive menstrual bleeding for the last week, associated with large clots. Detailed history revealed the presence of a facial rash, Raynaud’s phenomenon, photosensitivity and weight loss. She had no personal or family history of bleeding issues.

**Fig. 1 (Limit, 1):**
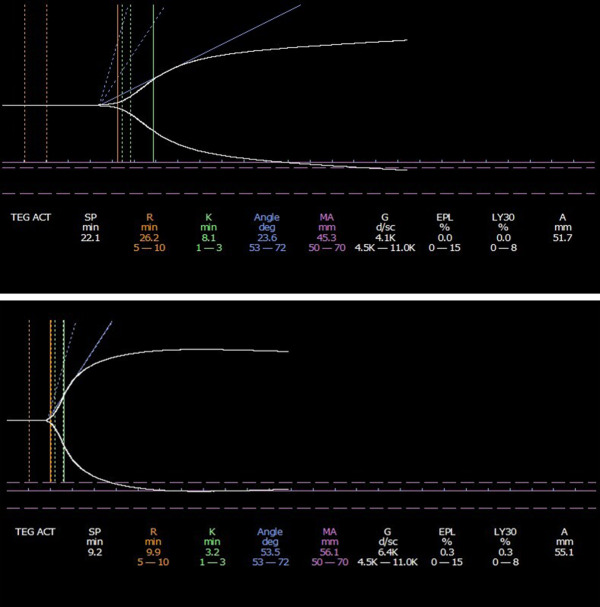
Top figure indicates TEG results on initial presentation which indicate a prolonged R and K time, decreased alpha angle. Bottom figure shows improving R, K and alpha values following 4 doses of rituximab

Vitals were stable. On physical exam, she was pale and had a facial maculopapular rash that spared the nasolabial folds with nasal ulceration. Labs on presentation were notable for: hemoglobin 6.3 g/dL, platelets 134 k/mm^3^, prothrombin time (PT) 21.2 s (above limit of normal reference interval, 13.0), activated partial thromboplastin time (aPTT) 46.3 s (above limit of normal reference interval, 37.0). Lactate dehydrogenase, haptoglobin and retic panel were within normal limits, ruling out hemolysis. Coombs test was also negative. The aPTT mixing study failed to correct into normal reference intervals after the addition of normal plasma and incubated at 37 degrees C. The patient plasma/normal plasma mixture APTT was run immediately, 1-hour, and 2-hour time points, indicating the presence of a high titer inhibitor. Initial testing for factors V, VII, VIII, IX, and X showed evidence of inhibition but corrected with serial dilutions. The Bethesda assay for FVIII inhibitor and the fibrinogen returned within normal reference intervals. Von Willebrand panel was also within normal reference intervals. Thromboelastography (TEG) study was significantly aberrant with prolonged R, K and alpha-angle indicating clotting factor deficiency (Fig. 1). Initial supportive management included several units of packed red blood cells, fresh frozen plasma, aminocaproic acid and hormonal therapy to suppress vaginal bleeding. Laboratory tests with a longer turn-around time came back a few days later. Clot based Lupus anticoagulant testing was positive against two separate anti-phospholipid sources. The method of testing is consistent with 2020 ISTH lupus anticoagulant detection guidelines where a three-step procedure with two test systems (diluted Russell’s viper venom time and aPTT) is recommended. Simultaneous performance of the mixing and confirmatory step, in each sample with a prolonged screening test was also employed. Additionally, the patient had high-titer IgM and IgG antibodies against Anti-cardiolipin (IgM > 112 MPL U/mL, IgG > 112 GPL U/mL; laboratory cutoff 0–23 U/mL) and B-2-Glycoprotein 1 (IgM > 112 M units, IgG > 112 G units; laboratory cutoff 0–20 units). According to the laboratory guidelines for Lupus Anticoagulant testing, the testing was consistent with “triple positivity” of antiphospholipid antibodies. Autoimmune laboratory evaluation also showed a high titer ANA (1:1280 by immunofixation; nuclear homogenous) and dsDNA (> 300 IU/mL by ELISA).

Clinical criteria of SLE were met, and thereafter she was started on treatment with steroids (oral prednisone, 40 mg daily) and hydroxychloroquine (200 mg daily). In the absence of thrombosis or pregnancy-related morbidity, she did not fulfil the criteria for antiphospholipid syndrome (APS) initially. She presented two weeks later with an SLE flare having fevers, arthralgias and pleuritic chest pain. In the course of its management, she also developed a left femoral deep venous thrombosis. Renal laboratory workup was as follows: Creatinine 0.66 mg/dL; urinalysis protein 1+; urine total random protein 120 mg/dL. In view of the recent diagnosis of SLE, a renal biopsy was deemed necessary which was complicated by excessive bleeding and a perinephric hematoma. Recombinant factor VIIa was used to establish hemostasis. By this time the diagnosis of SLE and secondary APS had been established but the underlying etiology of her coagulopathy and bleeding was yet to be identified. Factor levels were repeated with serial dilutions, all of which were normal except Factor II which was significantly low at 14% and did not correct with serial dilution. She was then diagnosed with lupus anticoagulant hypoprothrombinemia syndrome (LAHPS).

Despite the use of methylprednisolone and hydroxychloroquine, her clinical course continued to worsen with multiple systemic symptoms and clinically significant bleeding and thrombosis. After a multidisciplinary discussion, rituximab, a recombinant anti-CD20 antibody was started. She received weekly rituximab at a dose of 375 mg/m^2^ in addition to another steroid pulse. PT and aPTT tests began to normalize within a week. After four weekly doses of rituximab, Factor II level and TEG assay completely normalized (Fig. 1 & supp. material). Hydroxychloroquine was continued. After more than 5 years of follow up, she has had no bleeding or thrombosis recurrence with a normal Factor II level. She, however, continues to have persistently positive triple positive APL and has been treated with daily aspirin therapy.

## Discussion

LAHPS is a rare entity and is a combination of antiphospholipid syndrome and an isolated acquired Factor II deficiency. The possible presence of prothrombin antibodies should be suspected if a patient with antiphospholipid antibodies develops bleeding rather than the expected thrombotic events [2]. Less than 100 cases have been reported in the literature so far [3, 4]. The bleeding manifestations can range from only bruising to catastrophic consequences like uncontrolled pulmonary hemorrhage and intracranial hemorrhage [5, 6]. It is most commonly seen in pediatric and young adult patient, with a female predilection and is predominantly associated with autoimmune disease such as SLE and acute viral infections [4].

In APS, antiphospholipid antibodies are directed against the predominant antigenic target, β2GPI. This is central to the pathogenesis of APS whereby the antibodies recognize β2GPI bound to the surface of endothelial cells, monocytes, and immobilized platelets leading to cellular activation and expression of procoagulant effect [7]. While the prolongation of aPTT that is commonly seen in APS is due to the interference created by the antiphospholipid antibodies with phospholipid dependent coagulation studies in-vitro, antiprothrombin antibodies in LAHPS prolong the PT with a completely different mechanism of action. The antiprothrombin antibodies are non-neutralizing and bind to prothrombin to make an antigen-antibody complex which is cleared rapidly by the reticuloendothelial system. The decrease in prothrombin results in a prolonged PT in vitro as well [3]. The interference from the antiphospholipid antibodies can make factor testing very challenging and may thus delay a definitive diagnosis. In our case, utilization of the TEG assay, especially prior to finalization of send-out testing, was quite instrumental in indicating the presence of a hypocoagulable state and clotting factor deficiency.

There is no established treatment for LAHPS. In the scenario where the condition manifests secondary to a transient trigger, like a viral infection or a drug, supportive treatment is usually sufficient and definitive treatment might not be warranted. In other instances where the antiprothrombin antibodies persist, some form of immunosuppression is the norm. Immunosuppression is expected to downregulate antibody production and the subsequent clearance of prothrombin from circulation [3, 8]. Supportive treatment includes transfusion of fresh frozen plasma and prothrombin concentrate to help replete some of the prothrombin which can help control bleeding [3]. However, the presence of antibodies lead to rapid clearance of the prothrombin and bleeding tends to recur in most cases. This was also seen in our case.

Corticosteroids are the first line immunosuppressants used in this condition. While they are usually effective, escalation to other immunosuppressive agents is sometimes needed. Rituximab, used in some severe and refractory cases of APS, was chosen in our case [9, 10]. The first case of successful use of rituximab in LAHPS was described in literature in 2007 by Raflores et al. [11]. They described its use in combination with plasmapheresis and steroids in a LAHPS patient in the preoperative setting. The benefit was not long-lasting, however. In another case of a 35-year-old woman with SLE and LAHPS, rituximab was added after prothrombin level failed to rise despite high-dose corticosteroids and intravenous immunoglobulins. She had an excellent response and displayed durable remission off steroids for many months [12]. To our knowledge, we identified one report detailing novel and successful use of rituximab in LAHPS in 3 pediatric patients [13]. More reports of its efficacy are necessary to build a robust body of evidence in favor of using it either as upfront or in instances when steroid refractoriness is encountered.

## Electronic supplementary material

Below is the link to the electronic supplementary material.

Supplementary Material 1: Prothrombin levels after introduction of Rituximab

## Data Availability

All data underlying the results are available as part of the article and no additional source data are required.
