# Supplementary figures and images for: Successful treatment of lupus anticoagulant hypoprothrombinemia syndrome with rituximab
Source: Thromb J. 2023 Jul 17;21:77. doi: 10.1186/s12959-023-00517-z (PMC10353230; doi:10.1186/s12959-023-00517-z)

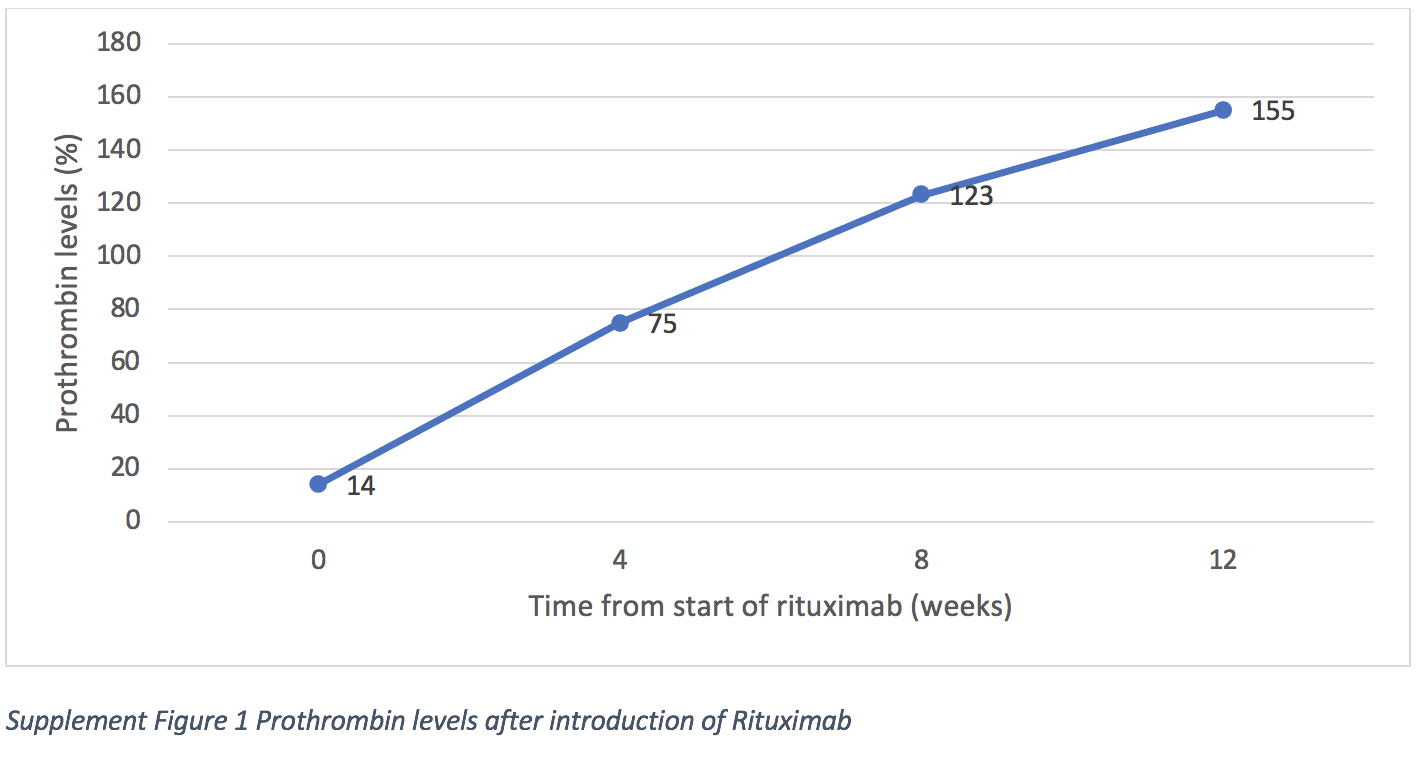

Supplement: Supplementary file 1 — Supplementary Material 1: Prothrombin levels after introduction of Rituximab [file 12959_2023_517_MOESM1_ESM.png]
